# An efficient method for markerless mutant generation by allelic exchange in *Clostridium acetobutylicum* and *Clostridium saccharobutylicum* using suicide vectors

**DOI:** 10.1186/s13068-019-1364-4

**Published:** 2019-02-14

**Authors:** Celine Foulquier, Ching-Ning Huang, Ngoc-Phuong-Thao Nguyen, Axel Thiel, Tom Wilding-Steel, Julie Soula, Minyeong Yoo, Armin Ehrenreich, Isabelle Meynial-Salles, Wolfgang Liebl, Philippe Soucaille

**Affiliations:** 1 LISBP, INSA, University of Toulouse, 135 Avenue de Rangueil, 31077 Toulouse Cedex, France; 2 Chair of Microbiology, Technical University Munchen, Emil-Ramann-Str. 4, 85354 Freising, Germany; 3 Tan Tao University, School of Medicine, University Avenue, Tan Duc e-City, Duc Hoa, Vietnam; 4 BBSRC/EPSRC Synthetic Biology Research Centre, School of Life Sciences, Centre for Biomolecular Sciences, University of Nottingham, University Blvd, Nottingham, NG7 2JE UK

**Keywords:** *Clostridium acetobutylicum*, *Clostridium saccharobutylicum*, *upp* gene, 5-FU, Restrictionless, Markerless, Gene deletion, Gene replacement

## Abstract

**Background:**

*Clostridium acetobutylicum* and *Clostridium saccharobutylicum* are Gram-positive, spore-forming, anaerobic bacterium capable of converting various sugars and polysaccharides into solvents (acetone, butanol, and ethanol). The sequencing of their genomes has prompted new approaches to genetic analysis, functional genomics, and metabolic engineering to develop industrial strains for the production of biofuels and bulk chemicals.

**Results:**

The method used in this paper to knock-out, knock-in, or edit genes in *C. acetobutylicum* and *C. saccharobutylicum* combines an improved electroporation method with the use of (i) restrictionless Δ*upp* (which encodes uracil phosphoribosyl-transferase) strains and (ii) very small suicide vectors containing a markerless deletion/insertion cassette, an antibiotic resistance gene (for the selection of the first crossing-over) and *upp* (from *C. acetobutylicum*) for subsequent use as a counterselectable marker with the aid of 5-fluorouracil (5-FU) to promote the second crossing-over. This method was successfully used to both delete genes and edit genes in both *C. acetobutylicum* and *C. saccharobutylicum*. Among the edited genes, a mutation in the *spo0A* gene that abolished solvent formation in *C. acetobutylicum* was introduced in *C. saccharobutylicum* and shown to produce the same effect.

**Conclusions:**

The method described in this study will be useful for functional genomic studies and for the development of industrial strains for the production of biofuels and bulk chemicals.

## Background

In recent years, solventogenic *Clostridia* have been of interest in the postgenomic era due to the complete sequencing and annotation of their genome [1, 2], supplying a wealth of information regarding the metabolism of these industrially important strains. This global knowledge has prompted new approaches to genetic analysis, functional genomics, and metabolic engineering to develop industrial strains for the production of biofuels and bulk chemicals.

To this end, several reverse genetic tools have been developed for solventogenic *Clostridia*, including gene inactivation systems based on nonreplicative [3–5] and replicative plasmids [6–10] and the group II intron gene inactivation system [11, 12]. All methods based on electroporation for *in frame* deletions use a replicative plasmid (typically containing a pIMP13 origin of replication from *Bacillus subtilis* that is functional in *Clostridia*) due to the low frequency of transformation of solventogenic *Clostridia* [13, 14]. Two families of methods have been developed to allow deletion and/or the introduction of genes at their normal chromosomal context without maintaining an antibiotic marker.

The first family [7, 10] uses a replicative vector containing (i) a replacement cassette consisting of an antibiotic resistance gene (*Th*^*R*^) flanked by two FRT sequences, (ii) two sequences homologous to the selected regions around the target DNA sequence, and (iii) a counterselectable marker made either of the codon-optimized *mazF* toxin gene from *Escherichia coli* (under the control of a lactose-inducible promoter) or the *upp* gene [which encodes an uracil phosphoribosyl-transferase and leads to 5-fluorouracil (5-FU) toxicity] to allow the direct positive selection of double-crossover allelic exchange mutants. After this first step, a second plasmid system expressing the FLP recombinase must be introduced, enabling efficient deployment of the FLP–FRT system to generate markerless deletion or integration mutants. A scar consisting of an FRT site remains at the target site, which can potentially act as a transcriptional terminator [15] or create a large chromosomal DNA deletion or inversion when several FRT sites are present on the chromosome [16, 17].

The second family [9, 18] also uses a replicative vector containing (i) a replacement cassette consisting of two sequences homologous to the selected regions around the target DNA sequence and (ii) a counterselectable marker made either of the *codA* [18] gene or the *pyrE* [9] gene. However, as the replacement cassette does not include an antibiotic resistance gene, and as this method uses a replicative plasmid, its stable single integration in the chromosome will be a rare event that cannot be selected for. When the counterselection is then applied, most of the clones will lose the plasmid and have a wild-type phenotype.

Creating a method for the rapid deletion, insertion, or modification of genes would require the use of a small suicide vector (to improve the transformation efficiency by electroporation), a replacement cassette consisting of two sequences homologous to the selected regions around the target DNA sequence and a counter selection marker such as *upp*, *codA,* or *pyrE*. One way to increase the transformation efficiency of solventogenic *Clostridia* is to remove the restriction modification system naturally present in the bacterium [5, 10, 14, 19]. Restrictionless, markerless mutants of solventogenic *Clostridia* have already been constructed for two species, *C. acetobutylicum* [10] and *C. saccharobutylicum* [5]. Although a transformation efficiency of 10^4^/μg DNA has previously been reported when using electroporation for a restrictionless mutant of *C. acetobutylicum* [10], the transformation efficiency of a restrictionless mutant of *C. saccharobutylicum* has not been measured [5].

In the present study, we further improved the transformation efficiency of the two restrictionless mutants by weakening the cell wall using a lysozyme treatment before electroporation. We then constructed small suicide vectors containing the *catP* or the *mls*^*R*^ genes for the selection of chromosomal integration and the *upp* gene to select, in combination with the 5-fluorouracil (5-FU) system, for the second crossing-over. These plasmids, the restrictionless strains with a *upp* deletion and the improved transformation protocol were successfully used to develop a method for gene knock-in, knock-out, and editing in *C. acetobutylicum* and *C. saccharobutylicum.*

## Results and discussion

### Transformation efficiency of different industrially relevant solventogenic *Clostridia*

In a previous study [10], we demonstrated that a restrictionless mutant of *C. acetobutylicum* could be transformed by electroporation with unmethylated pCons2.1 at very high efficiency (6 × 10^4^ transformants/μg of unmethylated DNA). However, when we evaluated the transformation efficiency of most of the non-sporulating, metabolically engineered strains, we noticed that the transformation efficiency of unmethylated pCons2.1 drastically decreased to values as low as 85 transformants/μg of unmethylated DNA. To improve the transformation efficiency of these industrially important, non-sporulating strains, we used as a prototype a *C. acetobutylicum* ∆*cac1502* ∆*cac3535*∆*upp*∆*pSOL* mutant that no longer sporulated or produced solvent. This mutant was obtained by spreading the *C. acetobutylicum* ∆*cac1502* ∆*cac3535*∆*upp* strain on an RCA plate and selecting clones that no longer produced a halo of starch hydrolysis after iodine staining [20]. The loss of pSOL1 was demonstrated by PCR analysis. The initial transformation efficiency of this strain with unmethylated pCons2.1 was low at approximately 142 ± 47 transformants/μg of unmethylated DNA (Table 1). Changing the voltage or the time constant did not significantly improve the transformation efficiency (data not shown). It was then decided to evaluate the use of cell wall weakening agents to facilitate DNA entry during the electroporation step. Such treatments, such as the use of lysozyme, have been shown previously [21–23] to improve the transformation efficiency of other Gram-positive bacteria. Lysozyme treatment, at concentrations ranging from 15 to 1500 μg/ml, was initially applied in the electroporation buffer for 30 min at 4 °C before electroporation. Although the transformation efficiency with unmethylated pCons2.1 was improved to values as high as 1 × 10^4^ transformants/μg of unmethylated DNA, the results were not reproducible. It was then decided to add the lysozyme treatment directly to the culture medium, before centrifugation and washing, according to the protocol described in “Methods”. Very reproducible results were then obtained with an optimal lysozyme concentration of 150 μg/ml resulting in a transformation efficiency of 6.5 × 10^3^ transformants/μg of unmethylated DNA, a value in the same range of the transformation efficiency of the sporulating *C. acetobutylicum* ∆*cac1502* ∆*cac3535*∆*upp* strain [10].

**Table 1:** Transformation efficiencies of *C. acetobutylicum* ∆*cac1502* ∆*cac3535* ∆*upp* ∆*pSOL* with unmethylated pCons2.1 plasmid

Lysozyme concentration (μg/ml)	Electroporation efficiencies
0	142 ± 47
15	648 ± 154
150	6.5 × 10^3^ ± 2.2 × 10^3^
1500	2.1 ± 0.2

Values are expressed in number of transformants per µg of unmethylated pCons2.1

Mean values and standard deviations from two independent experiments are given

5 µg pCons2.1 was used in each experiment

**Fig. 1:**
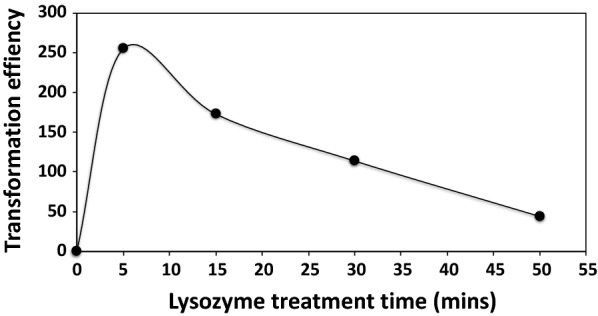
Effect of lysozyme treatment on the transformation efficiency the *C. saccharobutylicum* Ch2 (∆*hsdR1*∆ *hsdR2* ∆*hsdR3*) strain. Lysozyme (at a concentration of 150 μg/ml) was added in the culture medium when the A600 reached a value of 0.6 (see “Methods”). Incubation time varies between 5 and 50 min. The time point at t = 0 min correspond to an experiment without lysozyme added

In a previous study [5], Ch2, a markerless, restrictionless mutant of *C. saccharobutylicum* was constructed using conjugation to introduce the suicide vectors and the *codBA* genes and 5-fluorocytosine as a counter selection method. When Ch2 was transformed by electroporation using the unmethylated pMTL84151 replicative plasmid, no transformant could be obtained (Fig. 1) using the classical protocol without lysozyme treatment. On the other hand, when the protocol with the lysozyme treatment (optimized for non-sporulating *C. acetobutylicum*, i.e., 30 min of lysozyme treatment) was used, a transformation efficiency of 115 transformants/μg of unmethylated DNA was obtained (Fig. 1). After optimizing the incubation time with lysozyme (5 min), the transformation efficiency could be further increased to 255 transformants/μg of unmethylated DNA (Fig. 1). The unmethylated plasmid pMTL84151 was also used to evaluate the transformation efficiency, using the optimized protocol, of the *C. saccharobutylicum* wild type, Δ*hsdR1*, Ch1, and Ch2 strains. No transformants could be observed in the wild type and Δ*hsdR1* strain. In contrast, the transformation efficiencies of the Ch1 and Ch2 strains using unmethylated pMTL84151 were 58 and 255 transformants/μg of unmethylated DNA, respectively (Table 2).

**Table 2:** Transformation efficiencies of different *C. saccharobutylicum* mutants with unmethylated pMTL84151 plasmid

*C. saccharobutylicum* strain	Electroporation efficiencies
WT	0
Δ*hsdR1*	0
Ch1	58 ± 4
Ch2	255 ± 117

Values are expressed in number of transformants per µg of unmethylated pMTL84151

Mean values and standard deviations from two independent experiments are given

20 µg pMTL84151 was used in each experiment

### A generic method for gene knock-out, knock-in, and editing in *C. acetobutylicum* and *C. saccharobutylicum*

**Fig. 2:**
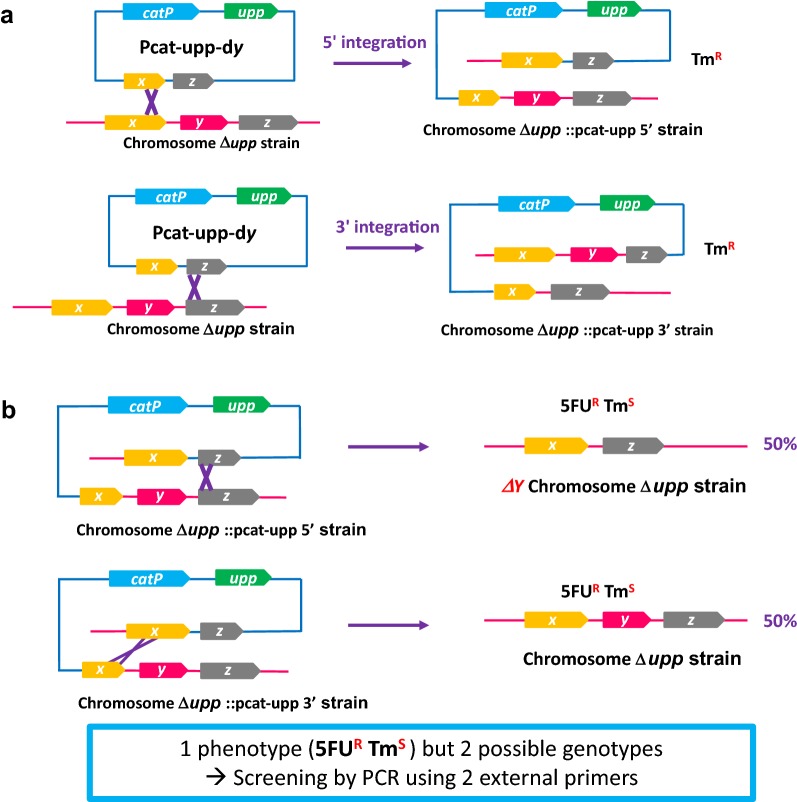
General diagram representing the generic method for gene modification by allelic exchange in solventogenic *Clostridia* using a restrictionless ∆*upp* strain. The example present the use of the method for markerless gene deletion, but it can also be used for markerless gene editing or DNA insertion. The gene to delete in frame is *y*. The boxed regions of *x* and *z* genes represent approximatively the regions of homology incorporated into the suicide plasmid. **a** Selection for plasmid integration in 5′ or 3′ using the antibiotic resistance carried by the suicide vector. **b** Counter selection strategy with the 5-FU/*upp* system used for the selection of the double crossing-over and the excision of the plasmid

To create the generic method (presented in Fig. 2) for gene modification in both species, two very small shuttle suicide vectors (pCat-upp and pEry-upp) were constructed that carry either a *colE1* or a *p15A* origin of replication functional in *E. coli*, a *upp* gene for 5-fluorouracil (5-FU) counterselection and either a *catP* or a *mls*^*R*^ gene for the selection of single crossing-over integration of the plasmid from thiamphenicol or erythromycin-resistant clones, respectively. Both plasmids have a unique *BamH*I site for the insertion of the modification cassettes.

**Fig. 3:**
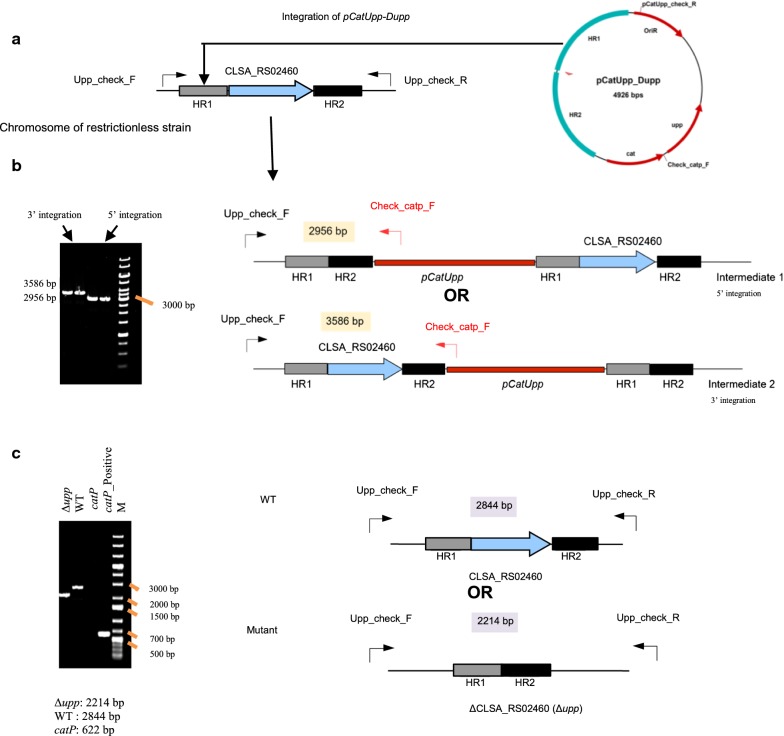
Markerless deletion of the *upp* gene in the *C. saccharobutylicum* Ch2 (∆*hsdR1*∆ *hsdR2* ∆*hsdR3*) strain. **a** Map of pCat-upp-Dupp and chromosomal region around *upp*. **b** Insertion of pCat-upp-Dupp in 5′ and 3′ of *upp*. Clones were characterized using the upp-check-F and check-catp-F primers. **c** Excision of the plasmid by a second crossing-over using 5-FU/upp as a counter selection tool and isolation of the *C. saccharobutylicum* ∆*hsdR1*∆ *hsdR2* ∆*hsdR3* ∆*upp* strain. All the 5FU-resistant thiamphenicol-sensitive clones had a *upp* deletion as demonstrated by PCR using upp-check-F and upp-check-R primers

The recipient strain should be restrictionless, but should also carry a *upp* deletion for counterselection using 5-FU. Such a strain was already constructed for *C. acetobutylicum* [10]. However, the Ch2 mutant of *C. saccharobutylicum* still had a functional *upp* gene. The pCat-upp-Dupp plasmid was then constructed by inserting in pCat-upp the *upp* deletion cassette containing two 1-kbp regions flanking the *upp* gene on the chromosome of *C. saccharobutylicum*. When Ch2 was transformed with 200 μg of this plasmid using the optimized electroporation protocol presented above, no clones resistant to thiamphenicol could be obtained. As such clones would result from a RecA-dependent crossing-over between the homologous regions of the plasmid and the chromosome, and as it is well known that RecA is more efficient on single-stranded DNA, the pCat-upp-Dupp plasmid was first denatured at 95 °C for 5 min and rapidly cooled on ice before electroporation. Applying this DNA pretreatment, approximately 10 thiamphenicol colonies were obtained. PCR analysis of the different clones showed (Fig. 3b) that integration was obtained both in the upstream and downstream regions of *upp*. Two clones with an integration in each homologous arm were grown in 2×YTG, and appropriate dilutions were plated on MES-MM (0.01% yeast extract) with 5-FU at 1 mM. To select integrants having excised and lost pCat-upp-Dupp, 5-FU-resistant clones were replica plated on both MES-MM (0.01% yeast extract) + 5-FU at 1 mM and 2xYTG with thiamphenicol at 15 µg/ml. To identify mutants that lost pCat-upp-Dupp and possessed a markerless *upp* deletion, clones resistant to 5-FU and sensitive to thiamphenicol (at 25 μg/ml) were checked by PCR analysis (with primers Upp-check-F and Upp-check-R located outside of the *upp* deletion cassette). All the 5-FU-resistant, thiamphenicol-sensitive clones showed that *upp* was deleted when analyzed by PCR (Fig. 3c). The fermentation profiles of one of the *C. saccharobutylicum* ∆*hsdR1*∆*hsdR2*∆*hsdR3*∆*upp* clones were evaluated in batch fermentation performed without pH regulation in MES-MM (0.001% yeast extract) medium. Solvent and acid formation by *C. saccharobutylicum* ∆*hsdR1*∆*hsdR2*∆*hsdR3*∆*upp* was similar to that of the wild-type strain (Table 3), indicating that no physiological modifications were introduced during the construction of the mutant.

**Table 3:** Solvent and acid formation by *C. saccharobutylicum* wild-type and mutant strains in batch culture without pH regulation

	*C. saccharobutylicum* wild type	*C. saccharobutylicum* ∆*hsdR1*∆*hsdR2*∆*hsdR3*∆*upp*	*C. saccharobutylicum* ∆*hsdR1*∆*hsdR2*∆*hsdR3*∆upp, spo0A*
[Acetone]_final_ (mM)	33	30	0
[Butanol]_final_ (mM)	83	76	0
[Ethanol]_final_ (mM)	11	9	6
[Acetate]_produced_ (mM)	11	15	28
[Butyrate]_final_ (mM)	12	16	47
Butanol yield (g g^−1^)	0.17	0.16	0

Cultures were done at 37 °C in MES-MM medium supplemented with 0.001% yeast extract for 96 h

### Gene deletion and editing in *C. acetobutylicum* using the generic method

**Fig. 4:**
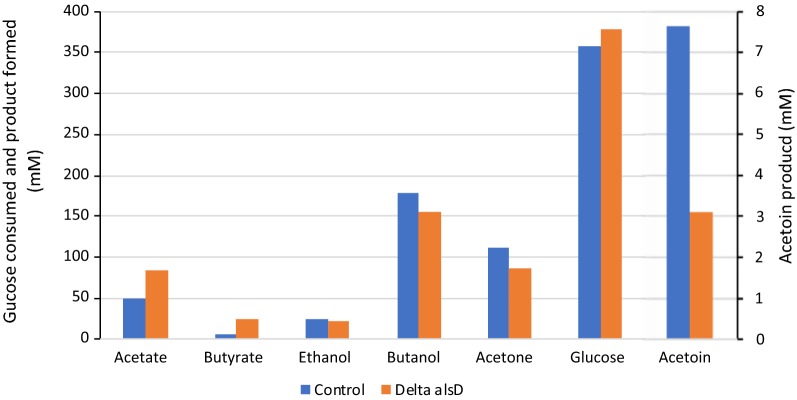
Solvent, acetoin and acid production by *C. acetobutylicum* ∆*cac1502* ∆*cac3535* ∆*upp* and *C. acetobutylicum* ∆*cac1502* ∆*cac3535* ∆*upp* ∆*alsD i*n batch culture at pH4.8 in SM medium. Cultures were ran for 72 h

The *alsD* gene (CA_C2967) encodes an acetolactate decarboxylase involved in the last step of acetoin formation [24]. To delete *alsD*, the *alsD* deletion cassette was cloned into the *Bam*HI site of the pCat-upp to generate the plasmid pCat-upp-alsD. The plasmid pCat-upp-alsD was used to transform the *C. acetobutylicum* ∆*cac1502* ∆*cac3535*∆*upp* strain by electroporation without previous in vivo methylation, and pCat-upp-alsD integrants were selected on RCA plates with thiamphenicol at 20 µg/ml. Two colonies were cultured for 24 h in liquid SM–glucose medium and then subcultured in liquid 2xYTG medium without antibiotic. Appropriate dilutions were plated on RCA with 5-FU at 1 mM. To select integrants having excised and lost pCat-upp-alsD, 5-FU-resistant clones were replica plated on both RCA + 5-FU and RCA with thiamphenicol at 40 µg/ml. To identify mutants possessing a markerless *alsD* deletion, clones resistant to 5-FU and sensitive to thiamphenicol were checked by PCR analysis (with primers alsd-0 and alsd-5 located outside of the *alsD* deletion cassette and primers alsd-F and alsd-R located inside *alsD*). Approximately half of the clones had an *alsD* deletion, and half had a wild-type genotype for *alsD*. The *C. acetobutylicum* ∆*cac1502* ∆*cac3535*∆*upp*∆*alsD* strain was isolated. The fermentation profile of this strain was compared to that of the *C. acetobutylicum* ∆*cac1502* ∆*cac3535*∆*upp* control strain during batch fermentation at pH 4.8 (Fig. 4). Surprisingly, the production of acetoin was only slightly decreased, indicating that either acetolactate can be chemically decarboxylated in vivo [25] or that Adc, the acetoacetate decarboxylase involved in the last step of acetone formation (15), can also decarboxylate acetolactate.

**Fig. 5:**
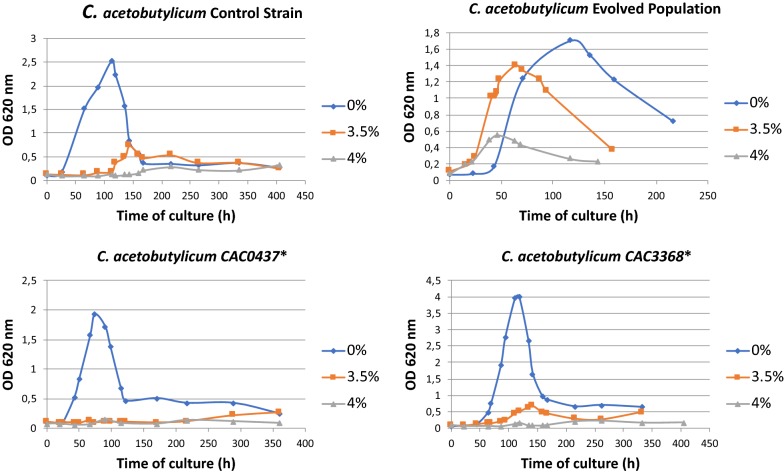
Isopropanol tolerance of various mutants of *C. acetobutylicum* ∆*cac1502* ∆*cac3535* ∆*upp*

In a project aiming to improve the isopropanol tolerance of *C. acetobutylicum* using an adaptive laboratory evolution (ALE) approach, three individual clones (IPT4, IPT7, and IPT10) able to grow at isopropanol concentrations higher than 40 g/l were isolated (Fig. 5). When the genomes of these three strains were sequenced, 26 mutations present in the three strains were identified. Among all the mutated genes, two retained our attention: CA_C 0437 and CA_C3368, which encode a phosphatase that catalyzes the dephosphorylation of Spo0A [26] and a putative permease, respectively. The mutation in each gene is translated at the protein level to C1151A and G506A mutations. To evaluate the effect of these mutations on isopropanol tolerance, the genome-editing method presented above was used to introduce each of the two mutations in the genome of *C. acetobutylicum* ∆*cac1502* ∆*cac3535*∆*upp.* For this purpose, two editing cassettes were created by directly amplifying a two kbp region centered around the point mutations in CA_C 0437 and CA_C3368 from the genome of the evolved strains and directly cloning them in pCat-upp to yield pCat-upp-CAC0437* and pCat-upp-CAC3368*.

Each plasmid was transformed by electroporation in the *C. acetobutylicum* ∆*cac1502* ∆*cac3535*∆*upp* strain and integrants were selected by their resistance to thiamphenicol. The generic method described in Fig. 2 was then used to select for the second crossing-over. Clones with the proper mutations were identified by a mismatch amplification mutation assay PCR (MAMA PCR) [27], and validation was finally performed by sequencing the region corresponding to the editing cassette plus 1 kbp on each side. The *C. acetobutylicum* ∆*cac1502* ∆*cac3535*∆upp::cac0437* and *C. acetobutylicum* ∆*cac1502* ∆*cac3535*∆upp::cac3368* were obtained and then characterized for their tolerance to isopropanol. The tolerance of both edited strains was not significantly different from the control strain (Fig. 5), indicating that those two mutations are either not involved in isopropanol tolerance or alone are not able to significantly participate in the isopropanol tolerance of *C. acetobutylicum*. Using the generic method described in this manuscript, a reverse strategy is currently under way, i.e., the editing back to wild type of each of the 26 mutations identified in one of the isopropanol tolerant strains and analysis of the isopropanol tolerance of the strains obtained.

### Use of the gene-editing method to assess the effect of the Spo0A G179S mutation on the control of sporulation and solvent formation in *C. acetobutylicum* and *C. saccharobutylicum*

During the selection process of the *C. acetobutylicum* ∆*cac1502* ∆*cac3535*∆*upp*∆*pSOL* strain, a mutant not producing solvent but still having the pSOL1 plasmid was identified and isolated. When the genome of this mutant was sequenced, a point mutation in the *spo0A* gene was identified, translating to the G179S mutation at the protein level. The mutated glycine residue is in a very conserved region of the Spo0A protein in all Firmicutes [28], IIHEI**G**VPAHI**K**GY, in which the lysine residue was shown to be involved in DNA binding to the Spo0A box [29].

**Fig. 6:**
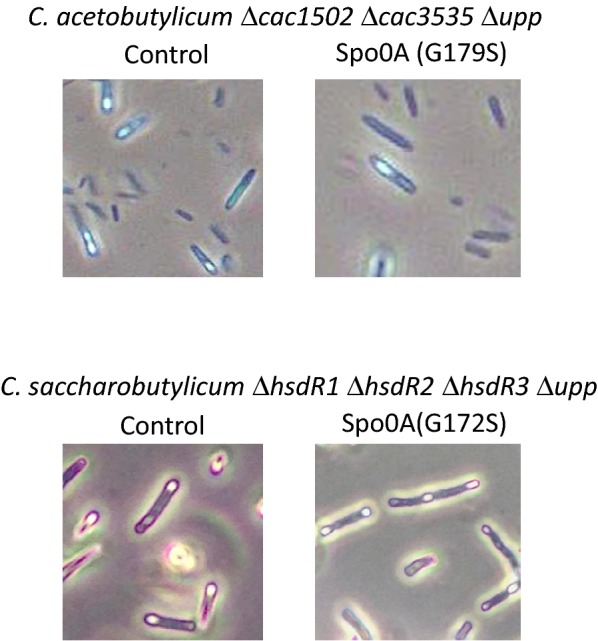
Sporulation of *C. acetobutylicum* ∆*cac1502* ∆*cac3535* ∆*upp* and *C. saccharobutylicum* ∆*hsdR1*∆ *hsdR2* ∆*hsdR3* ∆*upp* with and without the mutation in Spo0A (G179S and G172S, respectively)

This mutant was still able to sporulate, although at a lower frequency (Fig. 6), but after classical heat shock (70 °C for 10 min), no colony forming units were obtained for the G179S Spo0A mutant, while 4 × 10^5^ CFU/ml were obtained for the control strain (Table 4). Analysis of the product profile of the mutant showed that it no longer produced solvents, and only acetic and butyric acid accumulated in the fermentation broth (Table 5).

**Table 4:** Heat resistance of spores from different *C. acetobutylicum* and *C. saccharobutylicum* strains

*C. acetobutylicum* ∆*cac1502* ∆*cac3535* ∆*upp*		*C. saccharobutylicum* ∆*hsdR1*∆*hsdR2* ∆*hsdR3* ∆*upp*	
Control strain	Spo0A G179S	Control strain	Spo0A G172S
4 × 10^5^	0	5 × 10^7^	0

96 h cultures (in MES-MM medium supplemented with 0.001% yeast extract for *C. saccharobutylicum* and SM medium for *C. acetobutylicum*) were heat treated at 70 °C for 10 min. Values are expressed in number of CFU per ml of culture

**Table 5:** Solvent and acid formation by *C. acetobutylicum* ∆*cac1502* ∆*cac3535* ∆*upp* and mutant strain in batch culture without pH regulation

	*C. acetobutylicum* ∆*cac1502* ∆*cac3535* ∆*upp*	*C. acetobutylicum* ∆*cac1502* ∆*cac3535* ∆*upp*, spo0A*
[Acetone]_final_ (mM)	57	0
[Butanol]_final_ (mM)	139	0
[Ethanol]_final_ (mM)	41	10
[Acetate]_produced_ (mM)	− 21	27
[Butyrate]_final_ (mM)	9	68
Butanol yield (g g^−1^)	0.21	0

Cultures were done at 37 °C in SM medium for 96 h

Using the gene-editing method, the same mutation in *spo0A* (translating to the G172S mutation at the protein level, as this protein is 7 amino acid residues shorter in N-terminal than the corresponding *C. acetobutylicim* protein) was introduced in the *C. saccharobutylicum* ∆*hsdR1*∆*hsdR2*∆*hsdR3*∆*upp* strain. This mutant was still able to sporulate (Fig. 6), but similar to the *C. acetobutylicum* G179S Spo0A mutant, it no longer produced solvent (Table 3), and the spores were thermally sensitive (Table 4). A *tdcR* knock-out mutant of *C. difficile* was previously shown to also produce heat-sensitive spores, which was associated with a lower expression of the SigE- and SigF-dependent sporulation genes [30].

## Conclusions

The restrictionless, markerless generic method for genome modification in *C. acetobutylicum* and *C. saccharobutylicum* is a simple and useful tool for research groups involved in functional genomic studies and for further metabolic engineering of these two industrially important strains. As a demonstration of the efficiency of the method, we deleted the *alsD* gene in *C. acetobutylicum* to better understand how acetoin is produced in this microorganism. Furthermore, using this method we successfully edited genes to better characterize how *C. acetobutylicum* can develop isopropanol tolerance through adaptive laboratory evolution. Finally, we identified a mutation (G179S) in the Spo0A protein that abolishes solvent formation in both microorganisms while still allowing sporulation, although the spores produced were heat sensitive. Compared to the CRISPR/Cas9 method, that due to the large size of the *cas9* gene imposes the use of replicative, this method allows the use of suicide vectors avoiding the step of plasmid curing that can be troublesome.

In the future, with the combined use of the pCat-upp and pEry-upp vectors developed in this study, it should be possible to simultaneously inactivate two genes in case each of the single knock-out mutants is not viable, while the double knock-out mutant is viable.

## Methods

### Bacterial strain, plasmids, and oligonucleotides

The bacterial strain and plasmids used in this study are listed in Table 6. The specific oligonucleotides used for PCR amplification were synthesized by Eurogentec (Table 7).

**Table 6:** Bacterial strains and plasmids used in this study

Strain or plasmid	Relevant characteristics	Source/references
Bacterial strains		
*E. coli*		
TOP10		Invitrogen
*C. acetobutylicum*		
*∆cac1502*∆*cac3535*∆*upp*	∆*CA_C 1502*∆*CA_C 3535*∆*CA_C 2879*	[10]
∆*cac1502* ∆*cac3535*∆*upp* ∆*pSOL*	∆*CA_C 1502*∆*CA_C 3535*∆*CA_C 2879*∆*pSOL1*	This study
∆*cac1502* ∆*cac3535* ∆*upp* ∆*alsD*	∆*CA_C 1502*∆*CA_C 2879*∆*CA_C 3535*∆*CA_C 2967*	This study
∆*cac1502* ∆*cac3535* ∆upp::cac0437*	∆*CA_C 1502*∆*CA_C 2879*∆CA_C 3535:: CA_C0437*	This study
∆*cac1502* ∆*cac3535* ∆upp::cac3368*	∆*CA_C 1502*∆*CA_C 2879*∆CA_C 3535:: CA_C3368*	This study
∆cac1502 ∆cac3535 ∆upp::spo0A*	∆*CA_C 1502*∆*CA_C 2879*∆CA_C 3535:: CA_C2071*	This study
*C. saccharobutylicum*		
∆*hsdR1*	∆*CLSA_RS02150*	[5]
Ch1 (∆*hsdR1*∆ *hsdR2*)	∆*CLSA_RS02150* ∆*CLSA_RS14125*	[5]
Ch2 (∆*hsdR1*∆ *hsdR2* ∆*hsdR3*)	∆*CLSA_RS02150* ∆*CLSA_RS14125* ∆*CLSA_RS04425*	[5]
∆*hsdR1*∆ *hsdR2*∆ *hsdR3* ∆*upp*	∆*CLSA_RS02150* ∆*CLSA_RS14125* ∆*CLSA_RS04425*∆*CLSA_RS02460*	This study
∆*hsdR1*∆ *hsdR2*∆ *hsdR3* ∆*upp*, spo0A*	∆*CLSA_RS02150* ∆*CLSA_RS14125* ∆*CLSA_RS04425*∆*CLSA_RS02460*, CLSA_RS26780*	This study
Plasmids		
pAN1	*Cm*^*r*^, ϕ3TI, *p15A* origin	[14]
pUC18	*Ap*^*r*^, *colE1* origin	Fermentas
pCR-BluntII-TOPO	*Zeo* ^*r*^ *Km* ^*r*^	Invitrogen
pCons2-1	*Cm*^*r*^, *repL*	[10]
pMTL84151	pCD6, *Cm*^*R*^	[5]
pCons::upp	*MLS*^*r*^ *upp*, *repL*	[10]
pCR4-TOPO-Blunt	*Ap* ^*r*^ *Km* ^*r*^ *Cm* ^*r*^	Invitrogen
pCat-upp	*Cm*^*r*^ *upp*, *colE1* origin	This study
pEry-upp	*MLS*^*r*^ *upp*, *p15A* origin	This study
pCat-upp-Dupp	*Cm*^*r*^ *upp*, *upp* deletion cassette for *C. saccharobutylicum*	This study
pCat-upp-alsd	*Cm*^*r*^ *upp*, *alsD* deletion cassette for *C. acetobutylicum*	This study
pCat-upp-spo0A*Csa	*Cm*^*r*^ *upp*, *spo0A* editing cassette for *C. saccharobutylicum*	This study
pCat-upp-cac0437*	*Cm*^*r*^ *upp*, cac0437* editing cassette for *C. acetobutylicum*	This study
pCat-upp-cac3368*	*Cm*^*r*^ *upp*, cac3368* editing cassette for *C. acetobutylicum*	This study

*Cm*^*r*^: chloramphenicol resistance; *Ap*^*r*^: ampicillin resistance; *MLS*^*r*^: macrolide lincosamide and streptogramin B resistance; *Zeo*^*r*^: zeomycin resistance; *repL*: Gram-positive origin of replication from pIM13

**Table 7:** Oligonucleotides used for PCR amplifications

Primer name	Oligonucleotide sequence
pcat-Upp-F	AAAAAGGATCCGTGAGCAAAAGGCCAGCAAAAGGCC
pcat-Upp-R	AAAAAAGGATCCGTGAGCAAAAGGCCAGCAAAAGGCC
p15A-F	AAAAGGATCCTTAATAAGATGATCTTCTTGAGATCGTTTTGGT
p15A-R	AAAAGTCGACGCGCTAGCGGAGTGTATACTGGCTTA
eryUpp-F	AAAAGTCGACTCTACGACCAAAAGTATAAAACCTTTAAGAACTTTC
eryUpp-R	TATTTTACATTCTTTATTTTTTATTTTGTACCGAATAATCTATCTCCAGCATC
upp-Teradhe2-F	GATTATTCGGTACAAAATAAAAAATAAAGAATGTAAAATAGTCTTTGCTTCATTATATTAGC
teradhe2-R	AAAAGGATCCAAGATAAAAAACAAGAGTAAAATGTAAAATAGTCTATGTGC
Upp-Csa-1	ATTATGGATCCCCTGGAATGAAATATAGACATTATGCTCC
Upp-Csa-2	GTCCCAAATAATCTACTCATTTCATTATTCCTCCAAAACTTATATTATC
Upp-Csa-3	GGAATAATGAAATGAGTAGATTATTTGGGACTAAATAATCTGATGCAAG
Upp-Csa-4	ATAATGGATCCCGCACCTGCAAACGTAGTTGTAG
Upp-Check-F	ACGACCAGGTGGAATTAC
Upp-Check-R	CTTCCACATGGCCAACTC
Alsd-Cac-1	AAAATGATCACACCACATACAATTGCATATC
Alsd-Cac-2	GGTGAAGAAAAATGTAAGAGTATCCTAGAAGTGGTTTC
Alsd-Cac-3	TACTCTTACATTTTTCTTCACCTCAAACCAATTTATG
Alsd-Cac-4	AAAATGATCACCTTATTCATAATAATATGCCTCC
Alsd-Cac-F	TTAGAAACACCATTAGCACCTATAAAGGCT
Alsd-Cac-R	CGGTTAAACTTTTAAAAAAAGATAGCGATG
CAC0437_BAM_F	ATTGGATCCCTTGGCTTGAATGTATCAATGGAATTAAC
CAC0437_BAM_R	AATTGGATCCCCTTGTGAAGTTTGTGGTGGTAGC
CAC0437_EXT_F	CGATATGATCCCTATAGCACACG
CAC0437_EXT_R	CCTATGGGAGGGAAATCAACTTG
CAC0437_MAMA WT_F	GTAATGCTAAGACACAATTTATGGGGAC
CAC3368_BGLII_F	ATTAAGATCTTAGAAGTAGGCCCCATCTGCC
CAC3368_BGLII_R	ATTAAGATCTGGAGCGGTTATGAGAGAAAGACC
CAC3368_EXT_F	CCTGAGCTTATGGTACTCTGAAAGG
CAC3368_EXT_R	CATCTTGAGGAGTGTATGGAGATGC
CAC3368_MAMA WT_F	TATAGGAAGGTTTATAAAGAATATCCAAC
CAC3368_MAMA _R	TCCAGAGTTTGGCGACTACAT
Spo0A-Csa-1	TTTTGGATCCTCAAATAATTATTTAATGTTCCATTAGATAC
Spo0A-Csa-2	ATATCCTTTAATATGTGCAGGTACACTGATTTCATGAATGATGCTTGTAA
Spo0A-Csa-3	TTACAAGCATCATTCATGAAATCAGTGTACCTGCACATATTAAAGGATAT
Spo0A-Csa-4	TAATAAGGATCCTCAGATCCTAGATTGTTAGAGAAAACAGGA
Spo0A-Csa-F	TTTGAAATATTTTTTTCTTCTAAATAACTTG
Spo0A-Csa-R	AACTTCTAAATCAAACTTCTGTTGGTTCTAAAAG
Check_catp_F	AACTATTTATCAATTCCTGCAATTCGTTTAC
Check_catp_R	GGTATTTGAAAAAATTGATAAAAATAGTTG
*pCat-Upp check_R*	TCGCCACCTCTGACTTG

Restriction sites used for the cassettes construction are underlined

### Culture and growth conditions

*Clostridium acetobutylicum* and *C. saccharobutylicum* were maintained as spores in (SM) and MES-MM (0.001% yeast extract) synthetic media, respectively, as previously described [31–33]. Spores were activated by heat treatment at 70 °C for 10 min. All *C. acetobutylicum* and *C. saccharobutylicum* strains were grown in anaerobic conditions at 37 °C in SM or MES-MM (0.001% yeast extract), in *Clostridium* growth medium (CGM) [34] in 2xYTG [35], or in reinforced clostridial medium (RCM) (Fluka). Solid media were obtained by adding 1.5% agar to the liquid media. Media were supplemented, when required, with the appropriate antibiotic in the following concentrations: for *C. acetobutylicum* and *C. saccharobutylicum*, erythromycin at 40 µg/ml and thiamphenicol between 15 and 25 µg/ml; for *E. coli*, erythromycin at 200 µg/ml, and chloramphenicol at 30 µg/ml. 5-Fluorouracil (5-FU) was purchased from Sigma, and stock solutions were prepared in DMSO.

### Selection of isopropanol tolerant *C. acetobutylicum* mutant strains

An isopropanol tolerant population was selected using an Adaptive Laboratory Evolution (ALE) strategy using serial subcultures in SM–glucose medium with increasing concentration of isopropanol up to 5% W/V. Individual colonies were then on SM–glucose plates containing 4% W/V isopropanol. 10 clones were then evaluated for their isopropanol tolerance in liquid culture and the three best ones were sent for genome resequencing.

### Analytical methods

Cell growth was monitored by measuring optical density at 600 nm (OD600). Solvent and acid production as well as glucose consumption in cell-free supernatant samples were determined based on high-performance liquid chromatography (HPLC) [36] using H_2_SO_4_ at 0.5 mM, as mobile phase.

### DNA manipulation techniques

Total genomic DNA from *C. acetobutylicum* and *C. saccharobutylicum* were isolated as previously described [35]. Plasmid DNA was extracted from *E. coli* with the QIAprep kit (Qiagen, France). Pfu DNA Polymerase (Roche) was used to generate PCR products for cloning, and Taq Polymerase (New England BioLabs) was used for screening colonies by PCR with standard PCR protocols employed for all reactions. DNA restriction and cloning were performed according to standard procedures [37]. Restriction enzymes and Quick T4 DNA ligase were obtained from New England BioLabs (Beverly, MA) and were used according to the manufacturer’s instructions. DNA fragments were purified from agarose gels with the QIAquick gel purification kit (Qiagen, France).

### Transformation protocol

Transformations of *C. acetobutylicum* and *C. saccharobutylicum* were conducted by electroporation according to the following protocol. A 10% inoculum of *C. acetobutylicum* or *C. saccharobutylicum* was grown in CGM up to A_600_ of 0.6. This culture was used to inoculate a serum bottle with 50 ml of 2×YTG. When the culture reaches A_600_ of 0.6, 100 µl of 8% NH_4_OH is added to the cultures before putting it on ice. In the normal protocol, developed for sporulating *C. acetobutylicum*, cells were then harvested by centrifugation at 4500*g* and 4 °C for 10 min and the culture resuspended in 10 ml of ice cold 0.5 M sucrose, 10 mM MES, pH6 (EPB). After a second centrifugation under the same conditions, the pellet is resuspended in 400 µl of EPB. Cells were chilled on ice for 1 min in a sterile electrotransformation vessel (0.4 cm electrode gap × 1.0 cm) and plasmid DNA (5–200 μg) dialysed against EPB buffer was added to the suspension keeping the total volume constant at 0.6 ml. A 1.8 kV discharge was applied to the suspension from a 25 μF capacitor and a resistance in parallel of 200 Ω using the Gene Pulser (Bio-Rad Laboratories, Richmond, CA). The cells were immediately transferred to 10 ml of prewarmed 2×YTG and incubated overnight at 30 °C prior to plating on 2×YTG with 20 μg/ml and 15 μg/ml thiamphenicol for *C. acetobutylicum* and *C. saccharobutylicum*, respectively.

For the poorly transformable strains, i.e., non-sporulating *C. acetobutylicum* and *C. saccharobutylicum,* a lysozyme (from chicken egg white, 7000 U/mg, Sigma-Aldrich) treatment (final concentration ranging from 15 to 1500 μg/ml) for 5 to 30 min was introduced immediately after cooling on ice the culture. This lysozyme pretreatment was optimized for both *C. acetobutylicum* ∆*cac1502* ∆*cac3535*∆*upp*∆*pSOL* (a restrictionless non-sporulating strain) and *C. saccharobutylicum* Ch2 (a restrictionless sporulating strain).

### Construction of pCat-upp

This plasmid contains a *colE1* origin of replication functional in *E. coli*, a *cat*P gene conferring resistance to thiamphenicol and chloramphenicol, the *upp* gene (encoding the uracil phosphoribosyl-transferase of *C. acetobutylicum*) and a unique *Bam*HI site for the cloning of the replacement cassette. This plasmid was constructed by PCR (Phusion) amplification of a 2845 bp fragment on the pCons::UPP plasmid DNA using oligonucleotides pcat-Upp-F and *Bam*HI-pCat-Upp-R. This fragment was digested by *Bam*HI and ligated. The pCat-upp plasmid (2829 bp) was obtained.

### Construction of pEry-upp

This plasmid contains a *p15A* origin of replication functional in *E. coli*, an *mls*R gene conferring resistance to erythromycin, a *upp* gene and a unique *Bam*HI site for the cloning of the replacement cassette. This plasmid was constructed in five steps.

1. PCR (Phusion) amplification of the *p15A* replication origin (P15A fragment) on the plasmid pAN1, with the primers p15A-F and p15A-R.
2. PCR (Phusion) amplification of the *MLS*^*R*^ (*Ery*^*R*^) cassette (EryUpp fragment) on the pSOS95-Upp plasmid with the primers eryUpp-F and eryUpp-R.
3. PCR (Phusion) amplification of the *adhE2* terminator (Teradhe2 fragment) on *Clostridium acetobutylicum* genomic DNA with the primers upp-Teradhe2-F and teradhe2-R.
4. PCR fusion (Phusion) of the “EryUpp” and “Term-B” fragments using the primers eryUpp-F and termadhe2-R to get the “EryUpp- Teradhe2” fragment.
5. Digestion by *Bam*HI and *Sal*I of the “P15A” with “EryUpp- Teradhe2″ fragments and ligation to get the pEry-Upp plasmid (2582 bp).

### Construction of pCat-upp-Dupp

Two DNA fragments surrounding the *upp*-encoding gene (*CLSA_RS02460*) were PCR amplified with the Phusion DNA polymerase with total DNA from *C. saccharobutylicum* as template and two specific couples of oligonucleotides as primers. With the couples of primers *Upp*-*Csa*-1–*Upp*-*Csa*-2 and *Upp*-*Csa*-3–*Upp*-*Csa*-4, 1045 bp and 1047 bp DNA fragments were, respectively, obtained. Both primers *Upp*-*Csa*-1 and *Upp*-*Csa*-4 introduce a *Bam*HI site, while primers *Upp*-*Csa*-2 and *Upp*-*Csa*-3 have complementary 5′ extended sequences. DNA fragments *Upp*-*Csa*-1–*Upp*-*Csa*-2 and *Upp*-*Csa*-3–cac-4 were joined in a PCR fusion experiment with primers *Upp*-*Csa*-1 and *Upp*-*Csa*-4 and the resulting fragment was cloned in the pCR4-TOPO-Blunt vector to yield pTOPO:upp. The *upp* replacement cassette obtained after *Bam*HI digestion of the resulting plasmid was cloned, at the *Bam*HI, site into pCat-upp to yield the pCat-upp-Dupp plasmid.

### Construction of pCat-upp-alsd

Two DNA fragments surrounding the *alsD* encoding gene (*CAC2967*) were PCR amplified with the Phusion DNA polymerase with total DNA from *C. acetobutylicum* as template and two specific couples of oligonucleotides as primers. With the couples of primers *Alsd*-*Cac*-1– *Alsd*-*Cac*-2 and *Alsd*-*Cac*-3–*Alsd*-*Cac*-4, 1010 bp and 1011 bp DNA fragments were, respectively, obtained. Both primers *Alsd*-*Cac*-1 and *Alsd*-*Cac*-4 introduce a *Bgl*I site, while primers *Alsd*-*Cac*-2 and *Alsd*-*Cac*-3 have complementary 5′ extended sequences that introduced an in frame deletion of *alsD*. DNA fragments *Alsd*-*Cac*-1– *Alsd*-*Cac*-2 and *Alsd*-*Cac*-3–cac-4 were joined in a PCR fusion experiment with primers cac-1 and cac-4 and the resulting fragment was cloned in the pCR4-TOPO-Blunt vector to yield pTOPO:alsD. The *alsD* replacement cassette obtained after *Bgl*I digestion of the resulting plasmid was cloned, at the *Bam*HI, site into pCat-upp to yield the pCat-upp-alsd plasmid.

### Construction of pCat-upp-spo0A*Csa

Two DNA fragments surrounding the point mutation introduced in the *spo0A-*encoding gene (*CLSARS02460*) were PCR amplified with the Phusion DNA polymerase with total DNA from *C. saccharobutylicum* as template and two specific couples of oligonucleotides as primers. With the couples of primers spo0A*-*Csa*-1–spo0A*-*Csa*-2 and spo0A*-*Csa*-3–spo0A*-*Csa*-4, 797 bp, and 1204 bp DNA fragments were, respectively, obtained. Both primers spo0A*-*Csa*-1 and spo0A*-*Csa*-4 introduce a *Bam*HI site, while primers spo0A*-*Csa*-2 and spo0A*-*Csa*-3 have complementary 5′ extended sequences which introduce the point mutation. DNA fragments spo0A*-*Csa*-1–spo0A*-*Csa*-2 and spo0A*–spo0A*-3–spo0A*-4 were joined in a PCR fusion experiment with primers spo0A*-1 and spo0A*-4 and the resulting fragment was cloned in the pCR4-TOPO-Blunt vector to yield pTOPO: spo0A*-*Csa*. The *spo0A* replacement cassette obtained after *Bam*HI digestion of the resulting plasmid was cloned, at the *Bam*HI, site into pCat-upp to yield the pCat-upp-spo0A*-*Csa* plasmid.

### Construction of pCat-upp-cac0437* and pCat-upp-cac3368*

Cassettes containing the desired mutations surrounded by 1 kb upstream and downstream were PCR amplified with the Phusion DNA polymerase using total DNA from an isolated evolved isopropanol tolerant *C. acetobutylicum* strain (IPT4) as template and a specific couple of oligonucleotides as primers. For the CAC0437 PCR, the primers CAC0437_Bam_F and CAC0437_Bam_R were used to introduce a *Bam*H1 site, whereas for the CAC3368 PCR, the primers CAC3368_*Bgl*II_F and CAC3368_*Bgl*II_R were used to introduce a *Bgl*II site. The resulting fragments were cloned into the pCR4-TOPO-Blunt vector to generate pTOPO::CAC0437 C1151A and pTOPO::CAC3368 G506A, respectively. The CAC0437 C1151A fragment obtained after *Bam*H1 digestion and the CAC3368 G506A fragment obtained after *Bgl*II digestion were cloned at the *Bam*HI site into pCat-upp to generate the pCat-upp-CAC0437* and the pCat-upp-CAC3368* plasmids, respectively.

### Mismatch amplification mutation assay (MAMA PCR)

Primers for MAMA PCR were designed as described in publication [27] from Cha et al. Briefly, in each PCR, a forward MAMA primer and a reverse primer were used in a PCR reaction to detect the desired mutation. The PCR fragment was only generated from the wild-type gene and not from the gene with the mutation at the location covered by the mismatch position on the MAMA primer. For the CAC0437 C1151A mutation detection, the CAC0437_MAMA WT_F and CAC0437_ext_R primers were used. For the CAC3368 G506A mutation detection, the CAC3368_MAMA WT_F and the CAC3368_MAMA _R were used.

### Mutants’ characterization

For each mutant strain, two clones of were systematically selected and their deletion cassettes sequenced after integration into the chromosome by double crossing-over.

## Abbreviations

5-FU: 5-fluorouracil
CGM: *Clostridium* growth medium
DMSO: dimethyl sulfoxide
FLP: flippase
FRT: flippase recognition target
*MLS*^*r*^: the macrolide lincosamide streptogramin B resistance gene
PCR: polymerase chain reaction
EPB: electroporation buffer
RBS: ribosome binding site
RCM: reinforced clostridial medium
SM: synthetic medium
MES-MM: synthetic medium supplemented with MES
*Th*^*R*^: thiamphenicol resistance gene
UPRTase: uracil phosphoribosyl-transferase
